# Malignant salivary gland tumors: agreement between fine needle aspiration biopsy, incisional biopsy and final histopathological diagnostic

**DOI:** 10.4317/medoral.26101

**Published:** 2023-12-27

**Authors:** William Harvey Machado de Sousa Lacerda-Oliveira, Gláucia de Sousa Carvalho, Breno Washington Joaquim de Santana, Weslay Rodrigues da Silva, Mariana de Albuquerque Borges, Lélia Batista de Souza, Ana Paula Veras Sobral, Leorik Pereira da Silva

**Affiliations:** 1 DDS student, Federal University of Campina Grande, Patos, PB, Brazil; 2 DDS, Federal University of Campina Grande, Patos, PB, Brazil; 3 DDS, Research collaborator, School of Dentistry, University of Pernambuco, Camaragibe, PE, Brazil; 4 DDS, MSc, PhD student, Postgraduate Program in Dentistry, School of Dentistry, University of Pernambuco, Recife, PE, Brazil; 5 MD, Staff Pathologist, Department of Pathology, Cancer Hospital of Pernambuco, Recife, PE, Brazil; 6 DDS, MSc, PhD, Titular Professor, Postgraduate Program in Oral Pathology, Dentistry Department, Federal University of Rio Grande do Norte, Natal, RN, Brazil; 7 DDS, PhD, Associate Professor, Postgraduate Program in Dentistry, School of Dentistry, University of Pernambuco, Recife, PE, Brazil; 8 DDS, MSc, PhD, Adjunct Professor, Oral Histopathology Service, Federal University of Campina Grande, Patos, PB, Brazil

## Abstract

**Background:**

Incisional biopsy is indicated for intraoral tumors, but it is a contraindicated surgical procedure for major salivary glands. To avoid complications and facilitate diagnosis, fine needle aspiration biopsy (FNAB) is a type of biopsy widely used for preoperative diagnosis in these glands.

**Material and Methods:**

The aim of this study was to analyze the agreement between the diagnosis by FNAB (major glands), incisional biopsy (minor glands) and histopathological analysis of the surgical specimen in salivary gland tumors from a database (medical records) of patients treated in a cancer treatment reference center in the Northeast region of Brazil.

**Results:**

The sample consisted of 110 cases, being 86 of them malignant tumors in major salivary glands (parotid gland=73; submandibular gland=13) and 24 cases in minor salivary glands (palate). The female gender was the most affected (57.3%), especially in patients over 60 years (42.7%). In the TNM classification, 41.8% of the cases were in T2 at the time of diagnosis, with most of the regional lymph nodes in N0 (85.5%) and 87.3% of the cases in M0. FNAB was able to identify malignant neoplasms in 68.6% of the cases (*n*=59), while incisional biopsy in palatal tumors obtained agreement of 75% of the cases (*n*=18). The analysis revealed that tumors classified as T3-T4 (*p*=0.012) showed greater agreement between pre- and post-surgical diagnosis.

**Conclusions:**

The results obtained in this study indicate that FNAB has similar accuracy to incisional biopsy in providing satisfactory preoperative diagnoses of malignant tumors in salivary glands.

** Key words:**Neoplasms, salivary glands, fine needle aspiration biopsy.

## Introduction

The salivary glands comprise four pairs of major glands: parotid, submandibular, sublingual and, recently described, tubarial - located in the nasopharynx (1), in addition to the minor salivary glands that are numerous and widely distributed in the mouth and oropharynx (2). The neoplasms of the salivary glands are uncommon, constituting about 3% to 6% of all tumors of the head and neck and are characterized by a great heterogeneity of histological characteristics, biological behaviors and clinical outcomes (3-5).

The prognosis and clinical outcome of these lesions depend on the affected gland, histological patterns, clinical staging and molecular profile. It is also relevant to consider the involvement of the facial nerve, fixation or invasion in skin, deep structures, large caliber vessels or spread to lymph nodes and distant sites (6). Thus, the survival of patients affected by salivary gland tumors is directly correlated with several factors and early diagnosis is essential for a better prognosis (2).

For the establishment of the therapeutic strategy, a correct diagnosis is essential. In this sense, incisional biopsy of major salivary glands is considered a controversial and contraindicated surgical procedure, given the susceptibility to trans and postoperative complications, especially infections, dehiscence of suture, bleeding, neuropathies and fistulas. Several studies have sought alternative methods for early diagnosis of these lesions, with specificity, precision, technical ease and minimal surgical trauma (7). Fine needle aspiration biopsy (FNAB) is a biopsy type that is widely used for preoperative diagnosis of palpable masses and deep lesions of difficult access. In head and neck, it is usually used for diagnosis in thyroid, lymph nodes, major salivary glands and other less accessible sites (8). In other hand, incisional biopsy is considered a gold-patter procedure for lesions in minor salivary glands.

Due to the importance of preoperative diagnosis and early diagnosis of head and neck cancer that may interfere with the clinical outcome and survival of patients, studies on the accuracy of diagnostic tools are relevant for clinical practice. Therefore, the purpose of this study was to analyze the agreement between the diagnosis by FNAB, incisional and final histopathological biopsy (analysis of the surgical specimen after resection) in malignant tumors of salivary glands in a reference center for cancer treatment in the Northeast region of Brazil.

## Material and Methods

The study is cross-sectional, descriptive and adopts the documentary analysis of information contained in the patients' medical records as the data collection strategy. To carry out this research, a database in which was conducted a survey of all cases of malignant neoplasms of salivary glands was used, derived from the Head and Neck Surgery Sector of an oncological hospital in the Northeast region of Brazil, from 2007 to 2018. For the present analysis, 110 medical records were selected from cases in which the patient presented malignant neoplasm in the salivary gland and performed FNAB or incisional biopsy at the hospital, followed by surgical treatment for resection. Unfortunately, because it is an oncology hospital, we did not access diagnostic data of benign neoplasms.

Nomenclature and criteria used to histopathologically classify the cases were based on the descriptions summarized by the WHO classification of salivary gland tumors (9,10). When in doubt about the diagnosis, a second pathologist was consulted. There were no disagreements related to the reclassification and no case was excluded.

Descriptive and quantitative data analyses were performed using the Statistical Package for the Social Sciences for Windows, v. 24.0 (SPSS, Inc., Chicago, IL, USA). Pearson’s chi-square test adopting a *p value* of ≤ 0.05 was used. The research project was approved by the Research Ethics Committee of the Pernambuco Cancer Hospital (Protocol no. 2.901.350 - CAAE: 91084518.8.0000.5205).

## Results

Most of the patients in the sample were female (*n*=63/57.3%), and 21 (19.1%) of them were up to 40 years old, 42 (38.2%) between 41 and 60 and most of the patients were over 60 years old (47/72.7%); most individuals were not smokers (53.6%).

Regarding the anatomical location of the tumors in this study, 73 (66.4%) cases were diagnosed in parotid, 13 (11.8%) in submandibular and 24 (21.8%) in palate. The parotid gland presented 18 cases of NOS adenocarcinoma, 20 cases of mucoepidermoid carcinoma, 11 cases of acinic cell carcinoma, 4 cases of adenoid cystic carcinoma, 6 cases of epithelial-myoepithelial carcinoma, 2 cases of myoepithelial carcinoma, 3 cases of salivary duct carcinoma, 2 cases of cystadenocarcinoma, 2 cases of clear cell carcinoma, 1 case of carcinoma ex pleomorphic adenoma, 1 case of lymphoepithelial carcinoma, 1 case of sebaceous carcinoma, 1 case of carcinosarcoma and 1 case of squamous cell carcinoma; the submandibular gland presented 6 cases of NOS adenocarcinoma, 2 cases of mucoepidermoid carcinoma and 5 cases of adenoid cystic carcinoma; and the palate presented 6 cases of NOS adenocarcinoma, 1 case of mucoepidermoid carcinoma, 9 cases of adenoid cystic carcinoma, 1 case of epithelial-myoepithelial carcinoma, 2 cases of myoepithelial carcinoma, 1 case of cystadenocarcinoma, 3 cases of polymorphous adenocarcinoma and 1 case of mucinous adenocarcinoma.

According to the Classification of Malignant Tumors (TNM), it was observed that 63.6% (n = 70) of the cases were in T1 or T2 and 36.4% (n = 40) in T3 or T4; most (n = 94/85.5%) did not present nodal metastatic involvement (N0), while 14.5% (*n*=16) presented it in some degree (N1, N2, N3); 87.3% (*n*=96) of the cases did not present distant metastasis and 12.7% (*n*=14) did. Given these data, it can be concluded that: 58.2% (n = 64) were staged in grades I and II and 41.8% (n = 46) in grades III and IV.

A total of 77.3% (*n*=85) patients suffered recurrence and, in relation to the treatment method, the association of surgery and radiotherapy (44.5%) was the most used, followed by only surgery (29.1%), surgery, radiotherapy and chemotherapy association (16.4%) and surgery plus neck dissection (8.2%); 2 people (1.8%) abandoned treatment.

When clinical variables were statistically associated with the agreement between diagnosis of malignant neoplasms, benign lesions and inconclusive diagnoses, it was observed that only the tumor size variable (T3-T4) revealed a statistically significant association with concordance in the diagnosis of malignant neoplasms (*p*=0.012 - Pearson’s Chi-square test); no statistically significant association was observed for the other variables. This data indicates that tumors of larger size (T3-T4) have a higher probability of agreement between the diagnoses by FNAB and by final histopathological analysis.

Table 1 compares the diagnoses of salivary gland tumors obtained by FNAB with the final diagnoses obtained later, through histopathological examination of the specimen obtained by surgical resection. The Table 2 compares the diagnoses of malignant tumors initially obtained by histopathological examination of incisional biopsy with the final diagnoses.

In the Table 3, when we compare the preoperative diagnosis of salivary gland tumors by FNAB to those obtained by incisional biopsy of the lesions, shows that FNAB was used in most cases and presented a concordance of 68.6% (*n*=59) in the diagnosis of malignant neoplasms, whereas incisional biopsies showed a 75% (*n*=18) concordance of diagnosis of malignant neoplasm.

**Table 1:**
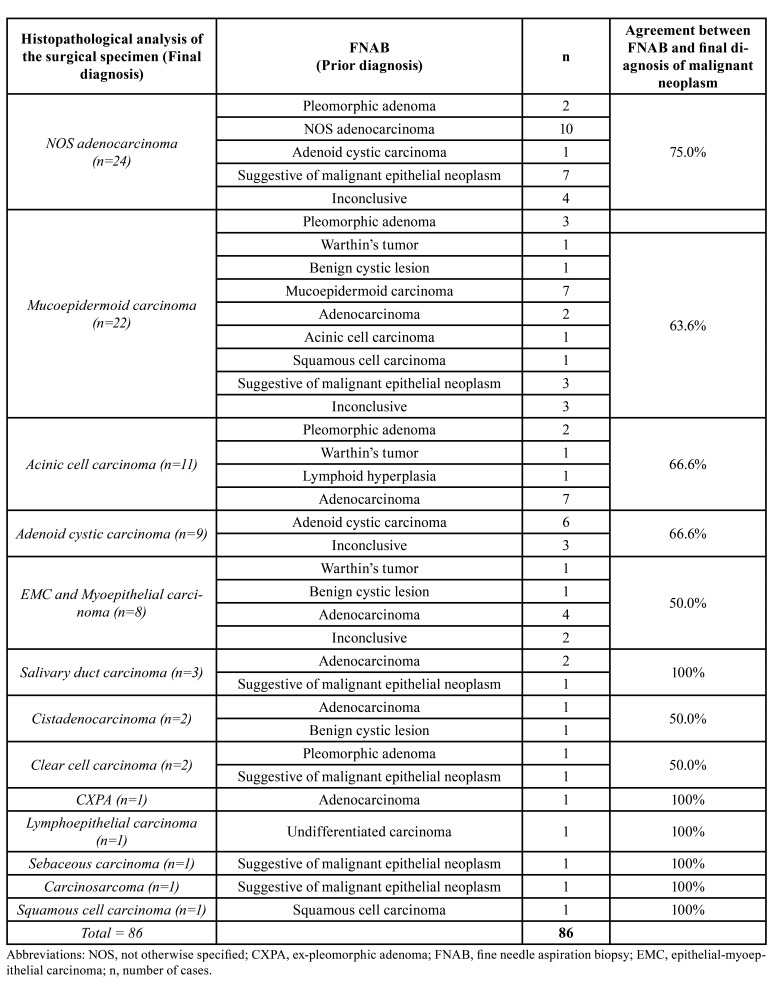
Comparative analysis of the malignant tumors diagnosis by FNAB and histopathological analysis of the surgical specimen in major salivary glands.

**Table 2:**
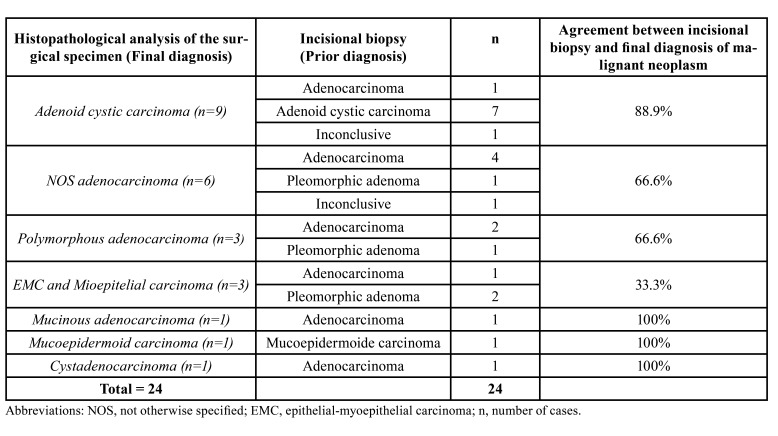
Comparative analysis of the diagnoses of malignant tumors by incisional biopsy and histopathological analysis of the surgical specimen in minor salivary glands.

**Table 3:**
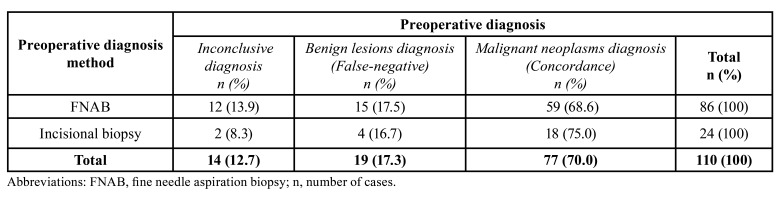
Comparative analysis of histopathological diagnoses of malignant tumors by incisional biopsy and fine needle aspiration in salivary glands.

## Discussion

The differentiation of salivary glands neoplasms in benign or malignant lesions from their clinical presentation is difficult, especially in early stages. Consequently, the diagnosis is based primarily on histopathological examination, which is a challenging process due to the sharing of morphological characteristics between distinct entities (11).

The Fine Needle Aspiration Biopsy (FNAB) is considered an easy, fast and economical method to obtain preoperative diagnoses in salivary glands (12) and has been used with the primary objective of differentiating non-neoplastic lesions from neoplastic - and differentiating benign from malignant lesions (13), since the treatment of both significantly differs. Although it has advantages, the ability of FNAB to provide an accurate diagnosis depends on some factors, such as the aspiration technique, sample size, material preparation, intratumoral heterogeneity and pathologist experience - in some cases, a definitive diagnosis is not possible even by experienced specialists (14).

This study only included cases of malignant final histopathological outcome. Of the 110 analyzed cases, the agreement (sensitivity) between the diagnosis obtained by FNAB and the final diagnosis of the surgical specimen was 68.8% and the percentage of false negatives was 17.5%. A similar study by İnançlı *et al*. (15) (2012), where the comparison between FNAB and final histopathological examination in the diagnosis of 115 salivary gland tumors was also performed, demonstrated that FNAB showed sensitivity of 80,8%, along with the fact that false negatives were 19.2% of the sample.

Other studies have shown that the sensitivity of this method widely varies from 42.9% to 90% (12-14,16-20), as in the study by Dostalova *et al*. (18) (2020), where through a retrospective analysis of medical records of 604 cases, the sensitivity of FNAB was 80% and false negatives were 20%, discarding the inconclusive samples. Several studies do not include insufficient samples for analysis in the final results, and one study showed that the proportion of inconclusive smears was higher in private establishments and that the level increased when it was done by training professionals (non-specialists) (12).

The analyzed study with the lowest sensitivity rate (42.9%) showed that, in cases where the FNAB presented low sensitivity, a greater rigor in the criteria of the cytopathologist was not enough for the correct diagnosis of some malignant lesions, highlighting the importance of the experience of the professional responsible for the examination (16). In the study with the highest sensitivity rate (90%), it was pointed out that the success was due to the low percentage of the categories "atypia of undetermined meaning" and "suspicion of malignancy" compared to those found in the literature, totaling 4.1% and 8.3%, respectively (20).

False-negatives (malignant lesions that were misdiagnosed as benign) ranged from 10% to 57.1% (12-14,16-20). One of the authors (17) does not indicate the routine use of FNAB in the evaluation of well-defined masses in the parotid, since the surgical plan or extension of resection do not change, and a possible false-negative result may dissuade the surgeon from a more indicated treatment modality.

Alternatively to FNAB and usually contraindicated in cases of tumors in major salivary glands, preoperative incisional biopsy is used in minor salivary glands lesions. Insufficient amount of material is also one of the pitfalls of this technique. In our study, the agreement (sensitivity) between the preoperative diagnosis by incisional biopsy and final diagnosis was 75%, very close to that found in the literature, although there are few studies that use this methodology. In a study by Fuoco *et al*. (11) (2022), the agreement between the incisional biopsy diagnosis and the final one of 56 gland lesions (benign and malignant) was 80%. In another, by Chen *et al*. (21) (2015), when they evaluated the agreement between these two methods in lesions at different sites of the oral cavity, it was found that in salivary glands-originated lesions, the result was 85.7%.

Finally, when we compare the findings of this study between rates of agreement obtained through FNAB and incisional biopsy, we have that the agreement obtained through the latter was higher (75%) when compared to that performed by puncture (68.5%), although the number of cases was lower. The rates of inconclusive diagnoses (13.9%) and false negatives (17.5%) were higher with the use of FNAB, with incisional biopsy values of 8.3% and 16.7%, respectively.

Both techniques have advantages and disadvantages. The FNAB has utility in the initial evaluation of lesions of major salivary glands and to confirm malignant lesions suspicion in order to plan and prognosticate the cases before the surgery or another form of initial treatment (11). One way to reduce the occurrence of inaccurate diagnoses in this method is to perform it with the aid of ultrasonic imaging instead of simply by palpation, which, according to some studies, confers higher sensitivity (18) due to greater chances of inserting the needle into solid areas of the lesion. The examination by expert and experienced professionals is also advised. Regarding incisional biopsy, studies show that multiple fragments revealed a greater tendency to "undetermined" diagnosis as it becomes difficult to analyze the patterns of growth and invasion of margins (11), being preferable only one fragment of a great extension.

Several discussions are observed regarding the clinical indication of FNAB in salivary gland tumors due possible histomorphological changes in tumors. However, in the study conducted by Díaz *et al*. (22) (2014), the authors highly recommend the use of FNAB in salivary gland tumors, and report significant indexes of sensitivity (94%) and specificity (100%). According to Table 4, the values of sensibility and specificity can vary among countries (12-14,16-20,22-28). It is a consensus that studies with benign and malignant tumors data are, undoubtedly, more complete to calculate the sensitivity and specificity of FNAB (22) since the sensitivity parameter measures the test ability to point to a malignant tumor result in people actually affected with these tumors, while specificity measures the test ability to point to a benign tumor result for cases that are, indeed, not malignant. Nonetheless, a limitation of the present study is that our data refer to records of an oncological hospital without data of patients with benign tumors.

Although FNAB is a safe diagnostic technique for salivary gland tumors, several nomenclatures and cytopathological classification methods can lead to misinterpretations, therefore, systematic diagnostic criteria are necessary to universally optimize the cytopathological diagnoses from FNAB of salivary glands. In this context, the system called "Milan System for Reporting Salivary Gland Cytopathology (MSRSGC)" is a collective work of the American Society of Cytopathology (ASC) and the International Academy of Cytology (IAC) to categorize the cytopathological diagnosis and correlate it to the recommended clinical management, as well as to the assumed risk of malignancy (ROM), making universal the communication between pathologists and clinicians and guiding the best therapeutic decisions (25,29). Our research did not initially use the MSRSGC because it is a retrospective study with archived reports dating from before the creation of Milan system and the cytopathologists involved used other methodologies.

**Table 4:**
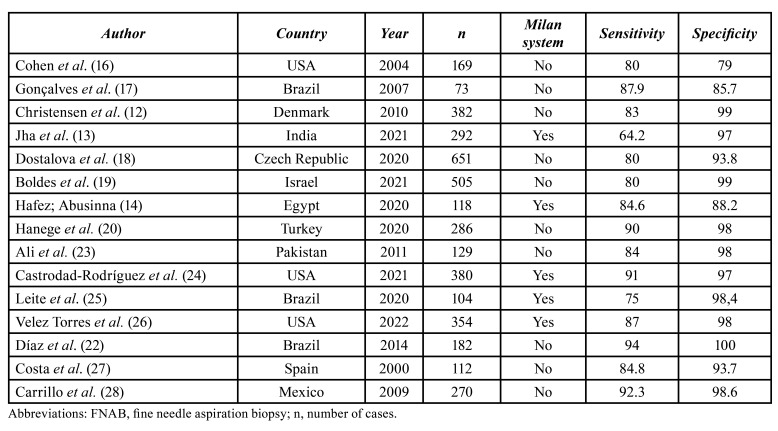
Variations of sensitivity and specificity of FNAB with and without Milan system.

However, when we categorized our sample according to Milan System (23,24), we found that: out of the 24 cases of NOS adenocarcinoma, 4 were classified as non-diagnostic, 2 as benign neoplasm, 7 as suspicious for malignancy and 11 as malignant; out of the 22 cases of mucoepidermoid carcinoma, 3 were classified as non-diagnostic, 1 as non-neoplastic, 4 as benign neoplasm, 3 as suspicious for malignancy and 11 as malignant; out of the 11 cases of acinar cell carcinoma, 1 was classified as non-neoplastic, 3 as benign neoplasm and 7 as malignant; out of the 9 cases of adenoid cystic carcinoma, 3 were classified as non-diagnostic and 6 as malignant; out of the 8 cases of epithelial-myoepithelial carcinoma/myoepithelial carcinoma, 2 cases were classified as non-diagnostic, 1 as non-neoplastic, 1 as benign tumor and 4 as malignant; out of the 3 cases of salivary duct carcinoma, 1 was classified as suspicious for malignancy and 2 as malignant; out of the 2 cystadenocarcinoma cases, 1 was classified as non-neoplastic and 1 as malignant; out of the 2 cases of clear cell carcinoma, 1 was classified as benign neoplasm and 1 as suspicious for malignancy; the case of carcinoma ex pleomorphic adenoma was classified as malignant; the case of sebaceous carcinoma was classified as suspicious for malignancy; the case of carcinosarcoma was classified as suspicious for malignancy; the case of squamous cell carcinoma was classified as malignant and the case of lymphoepithelial carcinoma was classified as malignant.

It should also be noted that the addition of oral and maxillofacial pathologists in the cytopathological and anatomopathological evaluation at hospital pathology services can contribute to the assertive diagnosis of diseases of salivary glands, since these professionals have expertise in diseases of the mouth and its adnexal tissues and structures.

## Conclusions

Fine needle aspiration biopsy (FNAB), the preferred biopsy modality in major salivary glands, is able to provide satisfactory preoperative diagnosis of malignant tumors in these organs, whereas incisional biopsies of minor salivary glands have better diagnostic accuracy by allowing the architectural morphological characteristics study that reflects the great diversity of subtypes of salivary gland tumors, data that are not obtained through a cytopathological analysis.
